# Clinico-Biological Implications of Modified Levels of Cytokines in Chronic Lymphocytic Leukemia: A Possible Therapeutic Role

**DOI:** 10.3390/cancers12020524

**Published:** 2020-02-24

**Authors:** Alessandro Allegra, Caterina Musolino, Alessandro Tonacci, Giovanni Pioggia, Marco Casciaro, Sebastiano Gangemi

**Affiliations:** 1 Division of Haematology, Department of Human Pathology in Adulthood and Childhood “Gaetano Barresi”, University of Messina, 98125 Messina, Italy; aallegra@unime.it (A.A.); cmusolino@unime.it (C.M.); 2 Clinical Physiology Institute, National Research Council of Italy (IFC-CNR), 56124 Pisa, Italy; alessandro.tonacci@ifc.cnr.it; 3 Institute for Biomedical Research and Innovation (IRIB), National Research Council of Italy (CNR), 98164 Messina, Italy; giovanni.pioggia@cnr.it; 4 Operative Unit of Allergy and Clinical Immunology, Department of Clinical and Experimental Medicine, University of Messina, 98125 Messina, Italy; mcasciaro@unime.it

**Keywords:** chronic lymphocytic leukemia, cytokine, immune system, B lymphocyte, T lymphocyte

## Abstract

B-cell chronic lymphocytic leukemia (B-CLL) is the main cause of mortality among hematologic diseases in Western nations. B-CLL is correlated with an intense alteration of the immune system. The altered functions of innate immune elements and adaptive immune factors are interconnected in B-CLL and are decisive for its onset, evolution, and therapeutic response. Modifications in the cytokine balance could support the growth of the leukemic clone via a modulation of cellular proliferation and apoptosis, as some cytokines have been reported to be able to affect the life of B-CLL cells in vivo. In this review, we will examine the role played by cytokines in the cellular dynamics of B-CLL patients, interpret the contradictions sometimes present in the literature regarding their action, and evaluate the possibility of manipulating their production in order to intervene in the natural history of the disease.

## 1. Introduction

### 1.1. General Considerations of Immunological Alterations in Chronic Lymphocytic Leukemia

B-cell chronic lymphocytic leukemia (B-CLL) is the main cause of mortality among hematologic diseases in Western nations [1]. It is a disease characterized by the proliferation of mature monoclonal B lymphocytes (CD19+/CD5+/CD23+ phenotype) in bone marrow, lymphoid tissue, and peripheral blood.

B-CLL is correlated with an intense alteration of the immune system. The altered functions of innate immune elements and adaptive immune factors are interconnected in B-CLL and are decisive for its onset, evolution, and therapeutic response [2].

T cells are augmented at the onset and may exert antitumor activity that influences disease development [3,4,5]. In fact, it has long been supposed that Th1 and Th2 cells are crucial antitumor cells in B-CLL, particularly the Th1 cells producing interferon gamma (IFN-γ). Moreover, several studies have confirmed that regulatory T (Treg) cells have a main role in tumor immune surveillance and tumor pathogenesis. However, neoplastic cells could directly stimulate Treg cells to deliver cytokines, forming an advantageous microenvironment for tumor proliferation and at the same time reducing the immune response. Numerous studies have demonstrated that Treg cells are augmented in CLL patients, and this would change the disease outcome [6,7,8,9]. Moreover, T cells from CLL patients display signs of exhaustion, presenting alterations in growth and activity [10], but the alterations of the immune system are decidedly more complex in B-CLL patients. B-CLL cells deliver practically all the cytokines [11,12], and cytokines may also originate from the relationship between B-CLL cells and T cells [13,14,15,16,17]. Modifications in the cytokine balance could support the growth of the leukemic clone, as some cytokines have been reported to be able to affect the life of B-CLL cells in vivo, by both the stimulation of growth and defense against programmed cell death [18,19].

Furthermore, the action performed by accessory cells should not be ignored. The more relevant evidence of the impact of accessory cells on B-CLL is the spontaneous programmed cell death of B-CLL cells cultured ex vivo. This phenomenon can be inhibited by the presence of cytokines and accessory cells. T cells, nurse-like cells, bone marrow stromal cells, dendritic cells, and, above all, the factors produced by them operate as pro-survival elements in B-CLL [20]. Moreover, the modification of lymphocyte and accessory cell subsets and the resulting change in the delivery of cytokines could also mediate the onset of infective complications, which is a frequent cause of death in B-CLL patients [21,22].

Finally, a modified lymphocyte balance and a specific cytokine signature would appear to be capable of changing the outcome of B-CLL. Patients with the progressive form of B-CLL had greater amounts of specific T cells that produced some cytokines compared to patients with a mild course of B-CLL.

In this review, we will examine the role played by cytokines in the cellular dynamics of B-CLL patients, interpret the contradictions sometimes present in the literature regarding their action, and evaluate the possibility of manipulating their production in order to intervene in the natural history of the disease (Table 1).

### 1.2. Effects of Cytokines on the Onset, Progression, and Complications of Chronic Lymphocytic Leukemia

Interleukin 2 (IL-2) is a four-*α*-helical bundle cytokine of 15.5 kDa size. It is essentially delivered by CD4+ T cells after an antigenic stimulus [70].

IL-2 works by binding to the IL-2 receptor (IL-2R). The connection of IL-2Rα (CD25), IL-2Rβ (CD122), and IL-2Rγ (CD132) elements give rise to a trimeric high-affinity IL-2Rα*βγ*. CD25 causes high affinity connecting to IL-2, while the β and γ subunits are responsible for the signal transduction. The presence of CD25 is indispensable for the growth of immunosuppressive Treg and natural killer (NK) cell growth and differentiation [71].

Musolino et al. demonstrated an increased plasma concentration of soluble CD8 (sCD8) and soluble IL-2R (sIL-2R) in B-CLL patients with respect to controls. The two antigen concentrations were significantly greater in patients with aggressive leukemia than in those with indolent B-CLL, and they also linked with Rai’s stage. Moreover, sIL-2R concentrations were related to lymphocyte count. These data seem to demonstrate that evaluating serum concentrations of sIL-2R may be useful to analyze the possible outcome of B-CLL [23].

Recently, an inhibitory receptor, Ig-like transcript 2 (ILT2), was identified, and its presence was significantly decreased on leukemic cells, while it was augmented on CD8 and CD4 T cells from B-CLL patients with chromosome 11q deletion, which involves the ataxia-telangiectasia-mutated kinase (ATM) gene. A significant modification of the expression of ILT2 ligands in B-cell chronic lymphocytic leukemia was also reported. CD4 and CD8 growth is downregulated by ILT2. Ig-like transcript 2 diminished the delivery of interleukin 2 by CD4 T-cells of B-CLL patients and provoked the generation of factors that is able to induce the persistence of leukemic cells by T cells. Notably, an ILT2 block re-established the cytokine production and growth of T cells [72].

The number of CD4+ and CD8+ T cells positive for IL-2 was reduced in B-CLL patients compared to controls [73], but IL-2 has protumoral activity. It has been demonstrated that IL-2 increases the growth of B-CLL cells in vitro. Moreover, an effect of IL-2 on spontaneous apoptosis has also been demonstrated. After 48 h culture, spontaneous apoptosis was blocked by 100 units/mL of IL-2 by 31.7% in B-CLL. This protective action of IL-2 against spontaneous apoptosis was dose-dependent [24].

IL-4 is a cytokine that provokes the differentiation of T cells to Th2 cells. After stimulation by IL-4, Th2 cells deliver supplementary IL-4 in a positive feedback loop. IL-4 is also produced by mast cells, NK-T cells, eosinophils, and basophils. IL-4 increases B-cell and T-cell growth, the maturation of plasma cells, and major histocompatibility complex (MHC)class II production [25,74,75].

The IL-4 pathway starts by connecting IL-4 to its receptor IL-4R. The activated IL-4R phosphorylates Janus kinase 1 and 3 (JAK1 and JAK3). JAK1 phosphorylates signal transducer and activator of transcription (STAT) 6, which goes in the nucleus to modify gene expression, with stimulation of the Ras/MAPK pathway and nuclear factor gamma-light-chain-enhancer of activated B cell (NF-κB) [76,77,78,79].

B-CLL cells show increased IL-4 gene expression, and this is one of the potential causes of increased concentrations of IL-4 in CLL patients. Numerous studies have demonstrated an IL-4 autocrine pathway in B-CLL lymphocytes, which act in programmed cell death [25,26,27].

IL-4 defends B-CLL cells from spontaneous programmed cell death or cellular death after DNA-injuring factors [28,29,30,31,32].

Regarding the mechanisms by which IL-4 can interfere with cell death, various hypotheses have been formulated.

Numerous studies demonstrated that adding IL-4 to cultured B-CLL cells augmented the stimulation of numerous STAT proteins that are able to stimulate several transcriptional elements that act in the survival of B-CLL cells [33]. Moreover, Ruiz-Lafuente et al. pointed out that miR-21-5p and other miRNAs held in the CLCN5 gene were connected to cytoprotection by IL-4 in CLL, suggesting that these RNAs are agents that are able to inhibit apoptosis [34].

B-CLL cells cultured with fibroblasts presenting CD40L and IL-4 (CD40L/IL-4) exhibited augmented protein synthesis [80,81]. In B-CLL cells, CD40L/IL-4 signal mediates the translational control of DNA damage repair genes, comprising ATM. Decreased concentrations of baseline ATM, independent of 11q deletion, are connected with decreased overall survival [82].

The Il-4 pathway could also be crucial in the onset of leukemia via mechanisms unconnected to apoptotic dynamics. In fact, several studies demonstrated that the immune alteration in B-CLL also involves a group of invariant natural killer T (iNKT) cells [83]. In fact, iNKT cells from patients with B-CLL displayed altered IL-4 and IFN-γ expression. The activity of iNKT cells was altered in patients with B-CLL by a relevant Th2 modification (high IL-4 and low IFN-γ expression). The ratio of iNKT + IFN-γ+ to iNKT + IL-4+ was reduced in B-CLL patients, and this diminished as the leukemia advanced. This alteration may provoke a stimulation of leukemic cell survival. Therefore, Th2 alteration may reduce the antitumor response that depends on stimulation of the Th1 immune response [35].

Finally, IL-4 would appear to also modify the therapeutic result in B-CLL patients. Therapy-naive patients with mutated B-CLL (M-CLL) have a tendency to respond less to ibrutinib treatment than patients with unmutated V-gene CLL (U-CLL) [84]. This distinction could be due to the fact that U-CLL patients have greater concentrations of surface immunoglobulin M (sIgM) expression and signaling ability compared to M-CLL patients [85]. IL-4 administration augmented sIgM expression on B cells from healthy controls and B-CLL patients, especially in patients presenting unmutated V-genes. The expression of sIgM induced by IL-4 caused an over-signaling of receptor activity, generating “mature” sIgM glycoform. In addition, IL-4 treatment reduced the anti-immunoglobulin M (IgM) blocking capability of idelalisib and ibrutinib, which are two BCR-associated kinase inhibitors. Finally, IL-4 reduced CXCR4 and CXCR5 expression; for this reason, unmutated CLL cells may diminish the characteristics of interleukin 4 to amplify B-cell antigen receptor (BCR) signaling and the retention of B-cells in the lymphonodes. As JAK3/STAT6 stimulate IL-4 activity, JAK inhibitors combined with BCR kinase inhibitors could have a role in therapy for B-CLL [86,87].

IL-6 is essentially produced by T cells and macrophages to intensify the immune response after infection [88]. IL-6 supports STAT3 and NF-κB stimulation via direct or indirect signals [89,90]. When stimulated, both transcription elements control the gene expression of factors implicated in the growth and persistence of tumor cells.

Nevertheless, STAT-3 is constitutively stimulated in B-CLL cells [91], and it was demonstrated that constitutively activated NF-κB in B-CLL cells caused the delivery of IL-6, which further increased JAK2/STAT3 stimulation [92]. IL-6/JAK/STAT3 established a feed-forward loop that controlled tumor proliferation [93].

IL-6 membrane-bound receptor CD126 was identified in all B-CLL samples and connected with the concentrations of STAT3 activity and chemoresistance treatment. Stopping CD126 with tocilizumab sensitized B-CLL cells to treatment [94].

IL-6 is recognized as being increased in B-CLL patients [95]. Augmented IL-6 serum concentrations were also demonstrated in advanced-stage B-CLL by Fayad et al. [96]. Nevertheless, it is uncertain if the origin of increased IL-6 is exclusively neoplastic B cells, as normal mononuclear cells are also recognized as being able to deliver IL-6 [97].

It has been reported that greater concentrations of IL-6 are connected with worse prognosis in B-CLL patients [96]. In fact, the higher the concentration of IL-6, the shorter the lymphocyte doubling time, time to first therapy, 17p/11q deletion, and progression-free survival. IL-6 could ameliorate B-CLL cells’ survival by activating STAT3 and NK-kB. The blockage of IL-6 or its receptor could alleviate the problem of B-CLL resistance to the treatment [36].

However, the mechanism of IL-6 in B-CLL is complex, and its activity in neoplastic angiogenesis has been recognized via modulation of VEGF expression [37,38], but the action of IL-6 is possibly diverse, depending on the cellular form and the microenvironment. In fact, other studies have demonstrated that IL-6 is also capable of blocking the proliferation of B-CLL cells [39].

To explicate the antiproliferative role of Il-6, various hypotheses have been formulated. Toll-like receptor (TLR) signaling is crucial in B-CLL biology, and in some cases, an action on TLRs may have a positive action on leukemia. For instance, the expression of TLR7 is limited to B-CLL cells, and the stimulation of TLR 7 by imidazoquinolines can re-sensitize B-CLL cells to treatment [98,99,100,101,102]. Moreover, IL-6 was observed to increase miR-17 and miR-19a, target TLR7 and tumor necrosis factor alpha (TNF-α) messenger RNA, and provoke a condition of tolerance to TLR7 agonists in B-CLL cells. The increased miR-17-92 cluster tolerized B-CLL cells directly and miR-17 and miR-19a antagomirs re-established TLR7 signaling. The blockage of IL-6 signaling with antibodies or Janus kinase inhibitors backed tolerization and augmented TLR7-stimulated B-CLL cell numbers. These data suggest that IL-6 may act as a leukemia suppressor by altering TLR signaling [40].

In conclusion, lacking exogenous TLR signaling, IL-6 augments short-term engraftment of B-CLL, while in the presence of TLR7 agonists, IL-6 operates as a leukemia suppressor by reducing its evolution. This study confirms the central role of the microenvironment in the fate of B-CLL cells [40].

IL-8, also recognized as CXCL8, is a pro-inflammatory chemokine of the CXC class. It was modified to produce a mature protein of 77 amino acids when it was delivered by parenchymal cells and 72 amino acids if it was delivered by monocytes and macrophages. The delivery of IL-8 was principally controlled by NF-κB transcription elements and by NF-IL6 [103].

IL-8 is one of the first cytokines described to act on B-CLL cells. Its serum concentrations are augmented in B-CLL patients compared to healthy controls, and it has been demonstrated that IL-8 increases B-CLL cell survival [104,105,106,107,108]. IL-8 participates via an autocrine modality to the B-cell accrual typical of this type of leukemia.

In vitro studies performed by Di Celle et al. reported that after the administration of IL-8, an increase of mRNA expression of bcl-2 by B-CLL cells happened [41]. As the increased bcl-2 is connected with reduced B-cell apoptosis, the increase provoked by IL-8 underlines the action of IL-8 in this leukemia [42].

In an old study, augmented concentrations of IL-8 were described in 25.8% of B-cell CLL patients. Levels of IL-8 did not show characteristics indicating tumor burden, such as b2-microglobulin, or bone marrow involvement. Nevertheless, circulating concentrations of IL-8 correlated with those of bcl-2, suggesting that the antiapoptotic action of IL-8 may be mediated by a bcl-2 pathway. Furthermore, it was more likely that stage A patients with concentrations of IL-8 above 4.5 pg/mL would evolve to a more advanced clinical stage [109].

Along with the action on programmed cell death, the effect of Il-8 on angiogenesis could be a different mechanism through which IL-8 exerts its protective effect on B-CLL cells [43,44].

However, not everything is clear regarding the relationship between IL-8 and B-CLL. In fact, it was found that this chemokine was lacking in blood samples [110] and lymph node samples from B-CLL patients [111]. Moreover, several studies reporting the capability of B-CLL cells to produce IL-8 ignored the fact that contaminating monocytes in B-CCL cell samples may be the real source of IL-8 production [112,113,114,115]. Then, without the real removal of monocytes from blood samples, it could have been possible to have false results concerning the capability of B-CLL cells to deliver IL-8.

Finally, CLL B cells do not appear to react to exogenous IL-8 when cultured alone or in the presence of monocytes/nurse-like cells [116].

Further studies seem necessary to define the role of IL-8 in the natural history of CLL.

IL-9 belongs to the family of the γ-chain cytokines. It uses the c-chain receptor and the IL-9 receptor (IL-9R), which is cytokine-specific [117,118]. IL-9 was first identified as a growth factor for T-lymphocytes, mast cells, and hematopoietic tumor cells [119,120,121]. The pro-proliferative and anti-programmed cell death action of IL-9 relies on its high-affinity connection with IL-9R. This link triggers the JAK/STAT pathways [122].

Several works reported that IL-9 is a factor that stimulates neoplastic proliferation, particularly lymphomagenesis [123,124]. In fact, even though IL-9 has been considered to be a Th2 cytokine that participates in allergic diseases, the latest results pointed out that IL-9 is implicated in tumor immunity regulated by mast cells and Tregs [125]. Its action in cell proliferation and in the programmed cell death of tumor cells indicates its role in tumor evolution, even if IL-9 has opposite effects according to the type of neoplastic cell [126]. As far as lymphoid cells are concerned, IL-9 is a cytokine that is able to stimulate the growth of lymphoma cells and defend them from dexamethasone-induced programmed cell death [45,46].

The altered expression of IL-9 evaluated at both the mRNA and protein levels has been reported in biopsies and in the serum of B-CLL patients [127]. This increase correlated with Rai staging, ZAP70, and CD38 [128].

These data were confirmed by other studies. In a report, IL-9 was identified in 20 of 47 serum samples from CLL patients, while no serum samples from control subjects contained detectable concentrations of cytokine. A greater expression of IL-9 was identified within (peripheral blood mononuclear cells (PBMCs) from B-CLL patients compared to controls, and the levels were correlated with β2 microglobulin expression and immunoglobulin heavy variable group (IgVH) status. These data indicate that increased IL-9 may participate in the onset of the disease and its dosage could be useful in prognostic evaluation [127].

Regarding the mechanisms by which IL-9 can cause the growth of B-CLL cells, it has been demonstrated that IL-9 stimulates the Janus kinase signal transducer and activator of transcription (JAK/STAT) pathway [46,47]. In fact, when adjoined with human IL-4 in culturing MEC-1 cells, levels of p-STAT6 and IL-9 in MEC-1 cells were augmented, and their production could be blocked by STAT6 inhibitor. Chen et al. demonstrated in B-CLL cells the existence of an extracellular IL-9/pSTAT3/microRNA-155 and -21/intracellular IL-9 positive feedback loop. IL-9 upregulation amplified the expression of pSTAT3, miR-155, and miR-21 in B-CLL. Moreover, a time-dependent increased production of IL-9 and rIL-9 caused STAT3 phosphorylation in MEC-1 cells. The use of WP1066 (a STAT3 inhibitor) stopped IL-9 production, notwithstanding the presence of rIL-9. Interleukin 9 levels were augmented in MEC-1 cells transfected by microRNA-21 and microRNAs 21–155 treated with rIL-9. WP1066 stopped MEC-1 cell apoptosis inhibition and growth induced by rIL-9 [48].

IL-10 is a cytokine delivered by helper T 2 cells, monocytes, and macrophages. It works as a negative feedback regulator of macrophage activity by inhibiting several cytokines, such as IL-1, IL-6, IL-8, and TNF-α [129]. The action of IL-10 in the onset and progression of B-CLL is still indeterminate. It has been reported that IL-10 increases DNA production and alters the migratory capacity of cells. IL-10 also cooperates with IL-2 for the growth of B-CLL cells by augmenting high-affinity IL-2 receptors [130].

The action of IL-10 in B-CLL programmed cell death is still uncertain. Bessler et al. demonstrated that there was a reduction of IL-10 levels in lymphocyte cultures from B-CLL patients [131], while other authors reported that serum IL-10 levels were increased in B-CLL patients, which may operate as an element to extend the lifespan B-CLL lymphocytes in vivo [96]. In fact, in Rai stage 2 and beyond, IL-10 blocks the programmed cell death of B-CLL lymphocytes [19].

The tight correlation between IL-10 and lymphocytes is manifested in B-CLL patients subjected to chemotherapy. Treg/Th17 and IL-10/IL-17 ratios in patients after chemotherapy compared with those before were diminished in the B-CLL patients in remission. In the non-remission group, Th17 cells and IL-17 were amplified and Tregs were lower, as well as IL-10 serum concentration; as a result, the IL-10/IL-17 proportion was diminished in the remission group. This ratio was linked to many B-CLL prognostic factors. An inverse correlation between Treg/Th17 and IL-10/IL-17 ratios and B-CLL progress was noticed, and this parameter was proposed as a marker of leukemia outcome. [132].

Several studies have attempted to clarify the mechanisms by which IL-10 could operate on the growth of B-CLL cells. IL-10 is a powerful immunosuppressor [133], and numerous reports have recognized IL-10-secreting B (B10) cells as potent immunosuppressive drivers accelerating the evolution of leukemia [134,135]. Actually, the number of B10 cells was augmented in EμTCL1-Tg animals and was connected with TCL1 expression [136].

Other research has been done on B-1 cells, which are a subset of B cells. It was demonstrated that B-1 cells and their malignant version, B-CLL cells, are capable of delivering IL-10. The more IL-10 is produced by B-1 cells, the more diminished their proliferation after BCR ligation. In fact, a significant reduction of B-1 cells is typical of animals that acquire a mutation of Bruton’s tyrosine kinase (Btk), which is a key enzyme of the BCR signaling pathway [137,138].

However, animals defective in Src homology region 2 domain-containing phosphatase-1 (SHP-1), cluster of differentiation 22 (CD22), or Sialic acid-binding immunoglobulin-type lectins (Siglec) G, which are proteins that negatively control BCR signaling, have augmented B-1 cells. Finally, the block of Src kinases, Syk, or Btk decreases IL-10 secretion by both normal and leukemic B-1 cells.

It was assumed that IL-10 could act in the control of the microenvironment of B-CLL in vivo by creating an immunosuppressive niche, allowing the leukemic cells to grow [139].

Finally, other possible pathways have been investigated. B-cell activating factor of the tumor necrosis factor (TNF) family (BAFF) is a crucial survival element essential for the growth of B2 B cells [140]. BAFF controls the programmed cell death of long-lived plasma cells residing in the bone marrow [141]. A paralogue of BAFF, a proliferation-inducing ligand (APRIL), also has pro-survival action on leukemic B-CLL cells. The autocrine delivery of BAFF in B-CLL patients is the main factor controlling leukemia progression [142]. It was described that BAFF signaling increased IL-10 production by leukemic cells in B-CLL patients [143].

Notwithstanding these results, not all the findings in the literature agree in confirming the negative action of IL-10 on B-CLL cells. Other studies demonstrated that IL-10 is able to increase the programmed cell death of B-CLL cells. Chong et al. [144] demonstrated that IL-10 reduced the production of p27 as a gatekeeper of the passage from G1 phase to S phase following programmed cell death with consecutive apoptosis initiation. IL-10 may provoke programmed cell death via the reduction of bcl-2 production in B-CLL cells. Moreover, IL-10 reduces the production of bcl-7c as a component of the anti-apoptotic Bcl family [145].

Finally, in an old study, IL-10 was inversely correlated with B-CLL progression [146].

IL-15 is part of a group of cytokines that also includes IL-2, IL-4, IL-7, IL-9, and IL-21 [147]. However, IL-15 has particular activities that are connected to its capacity to bind the a-chains of IL-15R [148]. The action of IL-15 is still unclear, but it is due to its transpresentation by cellular partners, such as dendritic cells, which are the principal origin of IL-15 in vivo [148,149,150,151].

IL-15 demonstrates relevant physiological actions in assisting with innate and adaptive immunity [152]. In fact, it has a central role in the stimulation of T lymphocytes and NK cells, increasing cytotoxic functions, stimulating IFN-g, TNF-a, and GM-CSF production, and regulating macrophage/NK relations [153,154]. Moreover, IL-15 is able to reduce the programmed cell death of neutrophils and eosinophils and Fas-mediated apoptosis of B or T cells via an increase of anti-apoptotic proteins [153,155].

Numerous studies discovered the role of IL-15 in autoimmune and inflammatory diseases and malignancies. These include hematologic diseases such as B-CLL. In this disease, IL-15 seems able to support the growth of leukemic cells in vitro [49,50].

In a study, Wu et al. demonstrated that recombinant human interleukin 15 (rhIL-15)-caused NK cell growth closely relied on an NK cell–B leukemic cell correlation through interleukin 15 receptor alpha subunit (IL-15Ra) [156].

IL-15 is a possible agent that is able to stimulate the TLR-9-triggered proliferation of B-CLL cells. Human memory B cells displayed strong in vitro growth after the administration of IL-15 and CpG DNA [157].

IL-15 blocks TLR-9-provoked programmed cell death and stimulates B-CLL clonal proliferation unrelated to the BCR mutation status.

A strong response to CpG oligodeoxynucleotide (ODN) + IL-15 was positively correlated with the presence of chromosomal anomalies and negatively correlated with the number of CD38+ cells in the B-CLL population, and the in vitro high-proliferator condition was correlated with reduced patient survival. These results, and the immunohistochemical finding of apoptotic cells and IL-15-generating cells near B-CLL pseudofollicles in B-CLL spleens, suggest that cooperative ODN and IL-15 signaling may stimulate in vivo B-CLL proliferation [158].

However, the existing data in the literature on the relationship between IL-15 and B-CLL are extremely contradictory, and IL-15 could be considered a new agent that is able to ameliorate the effect of immunotherapy.

In B-CLL, antibody-dependent cellular cytotoxicity is altered by the ratio of NK cells to B leukemic cells. Then, any treatment that is able to provoke augmented NK cells could be useful for B-CLL therapy. Laprevotte et al. studied the action of rhIL-15 on autologous NK cells in B-CLL samples. They demonstrated that rhIL-15 caused NK cell stimulation and growth, causing a B leukemic cell reduction. This was augmented in the presence of an anti-CD20 monoclonal antibody. Moreover, the superior action of obinutuzumab versus rituximab indicated collaborative action between rhIL-15 signaling and CD16 signaling in the simulation of NK cell growth. NK-rhIL-15-related growth is intimately linked to NK contact with B-CLL cells, which was identified as an additional element for rhIL-15 transpresentation. As reported, rhIL-15 gives a start to NK cell-based activity in B-CLL antibody immunotherapy [51].

Some researchers observed that rhIL-15 could diminish the immunosuppressive capability of TGF-beta in B-CLL models, provoking a superior rituximab (RTX)-mediated antibody dependent cell cytotoxicity (ADCC) [52]. In vivo and in vitro B-CLL samples had high levels of TGF-beta [159,160]. On this basis, TGF-beta seems to be involved in immune disequilibrium in B-CLL. Moreover, this mediator blocks the production of IFN-gamma mediated by CD16 as well as ADCC in NK cells in healthy subjects [161]. Potentially, administering rhIL-15 to patients affected by B-CLL could boost cytotoxic activity and reduce the immunosuppressive effect of TGF-beta on NKs. These effects could potentiate chemotherapy and the efficacy of monoclonal antibody therapy [162].

IL-17A is an inflammatory cytokine that is able to cause the delivery of IL-6 and IL-8 in numerous types of cells [163]. The main origin of the cytokine is Th17 cells [164,165].

Sherry et al. described substantial CD4 T cells with greater concentrations of IL-17F in patients affected by B-CLL compared to normal blood mononuclear cells after in vitro stimulation while exposed to Th17-promoting cytokines. Moreover, the progression of IL-17F-presenting Th17 cells was significantly amplified when purified CD4 T cells from B-CLL patients were cultured together with autologous B-CLL cells. Ultimately, single-cell network profiling confirmed that IL-17F augmented NF-kB phosphorylation in T and B cells from B-CLL patients. All of these findings indicate that the phenotype of Th17 cells in B-CLL patients is different from that in healthy subjects, presenting greater concentrations of IL-17F, and that lymphocytes from B-CLL patients are intensely reactive to IL-17F compared to healthy subjects [166].

Moreover, increased concentrations of IL-17 are connected with a worse clinical outcome [167]. 

Several suppositions have been formulated to explain the protumoral action of the cytokine. Bone marrow mesenchymal stem cells (BMMSCs) sustained the growth of B-CLL cells in vitro via an IL-6-dependent system. IL-17, which stimulates IL-6 production in several types of cells, augmented the generation of IL-6 in both B-CLL cells and BMMSCs. In an experimental model of B-CLL, BMMSCs and the culture supernatant augmented the growth of B-CLL cells via an IL-6-mediated mechanism. IL-17 administration also augmented B-CLL cell growth in mice with the same mechanism. The plasma of B-CLL patients presented increased concentrations of both IL-6 and IL-17 with respect to normal controls, and concentrations of IL-6 were correlated with IL-17 concentrations. B-CLL patients requiring fludarabine-based treatment presented greater concentrations of IL-6 and IL-17. These findings indicate the relevant action of the IL-17/IL-6 axis in B-CLL, which could be a useful therapeutic target [53].

Nevertheless, the action of IL-17 and its influence on IL-6 production in the evolution of B-CLL is ambiguous. Other researchers have asserted that Th17/IL-17 has protective action and that B-CLL patients with advanced-stage disease have minor concentrations of Th17/IL-17 [168], and Th-17 and IL-17A levels were reduced in patients with adverse prognostic factors. B-CLL patients with measurable IL-17A mRNA in T cells were in Rai stage 0 and negative for the expression of both Zeta-chain-associated protein kinase 70 (ZAP-70) and CD38. These data led the authors to speculate that Th17 could have a positive effect on B-CLL immunity [54].

IL-21 is a cytokine that belongs to the IL-2 family. It exerts its action through its receptor IL21R, which acts as a heterodimer with the common gamma chain [169,170].

IL-21 delivery was initially believed to be restricted to CD4+ T cells, but actually, we know that it is additionally generated by Th17 and NKT cells. Furthermore, IL-21 mRNA expression was reported in stromal cells in lymph nodes [171,172,173]. In any case, IL-21R has been reported to be present on B-CLL cells, and the presence of IL-21R seemed to be inversely correlated with CD38 expression but not ZAP-70 or mutational status of IgVH genes [55,174].

According to some authors, monocytes with IL-21 originating from lymph nodes were identified, whereas resting or CD40-activated B-CLL cells had no trace of IL-21 mRNA and protein [175]. These data suggest that IL-21/IL-21R might have a downgrading action in B-CLL. There are some experimental data that can confirm this hypothesis.

De Cecco et al. verified the capacity of IL-21 to control gene and miRNA expression in CD40-activated B-CLL cells. IL-21 was the main controller of chemokine delivery in B-CLL cells, and it regulated genes involved in programmed cell death, cell persistence, and cellular metabolism. In particular, IL-21 reduced the expression of CCL2, CCL3, CCL3L1, CCL4, and CCL17 genes, while it increased the expression of Th1-related CXCL9 and CXCL10. It was also reported that IL-21 reduced the expression of genes encoding signaling components, such as DDR1, CD40, and PIK3CD. IL-21 controlled the same genes in B-CLL and normal B cells. However, other genes, comprising TNF, Early growth response protein 2 (EGR2), MYC, E2F1, and Growth arrest-specific 6 (GAS-6), were controlled only in B-CLL cells. Then, numerous miRNAs were found to be controlled by IL-21, and these could regulate the expression of several target genes. These findings suggest that IL21 controls the expression of genes regulating the relationship between B-CLL cells and the microenvironment [56].

IL-21 treatment causes augmented perforin and granzyme B expression on CD8+ T cells, which could provoke the programmed cell death of B-CLL cells [57,58].

IL-21 has been demonstrated to have cytotoxic action on B-CLL cells. Nevertheless, the presence of IL21R differs significantly among B-CLL patients. This finding is relevant because, as reported above, receptor expression is connected with the apoptotic effect of IL-21 in B-CLL cells [55]. IL-21R receptor expression correlates with the proapoptotic BH3 domain protein BIM. IL-21-provoked BIM upregulation is essential for programmed cell death. The reduction of BIM expression by the use of small interfering RNA blocked IL-21-induced programmed cell death. IL-21 treatment of B-CLL cells with rituximab or fludarabine increased the cytotoxic action of these drugs. Moreover, IL-21 stimulated STAT1 and STAT5 phosphorylation in NK cells with concomitant increased antibody-dependent cytotoxicity against rituximab-coated B-CLL cells. These findings justify the possibility of using IL-21 with fludarabine and rituximab in B-CLL patients [174].

Numerous other studies have led to reports on the positive effects exerted by IL-21 on B-CLL cells. Neoplastic cells reacted to IL-21 stimulus by starting programmed cell death, and their responsiveness to IL-21 action was increased after being stimulating with CD40, CpG oligonucleotide, or CpG ODN. Moreover, increased IL-21R was also demonstrated after the stimulation of these factors [59].

IL-21 stimulated diverse JAK/STAT signals and increased programmed B-CLL cell death. In inactive B-CLL B cells, signaling via STAT3 was noticeably manifest, while signaling through JAK1 and STAT1 was scarcely evident. Another study confirmed that IL-21 may stimulate both STAT1 and STAT3 and provoke programmed cell death. In CD40-stimulated B-CLL cells, IL-21 activated JAK1 and JAK3, with the consequent phosphorylation of STAT1, STAT3, and STAT5, and it increased the programmed cell death of B-CLL cells.

Browning et al. reported that treating B-CLL cells with the ODN CpG-685 caused the overexpression of the IL-21 receptor. This elevation increased the effects of IL-21 by significantly affecting JAK1, STAT1, and STAT3 phosphorylation compared to IL-21 therapy without activating CpG preventively. The CpG-685 effect on IL-21R also augmented IL-21-induced cell toxicity. The exact mechanism of IL-21R overexpression is still not well described, although the mechanism of IL-21R in controlling T cells was correlated with the binding of the specificity protein 1 (Sp1) to the IL-21R promoter in T cells. It was reported that luciferase reporter constructs enclosing the Sp1 binding site had increased basal luciferase activity compared with constructs lacking the Sp1 binding site, but they were defective in increasing luciferase activity after CpG-685 stimulus in B-CLL cells. The CpG ODN-mediated induction of IL21R was diminished after the use of an NF-κB inhibitor, demonstrating that CpG-685 boosts IL21R via an NF-κB-mediated mechanism. These data indicate an alternative system for the stimulation of IL-21R in B-CLL cells and offer a basis for the conception of new combination treatments [60].

Finally, IL-21 stimulates the proliferation of cytotoxic T cells and suppresses Tregs, thus increasing T cell response. The use of IL-21 and CpG-685 could also ameliorate the patient’s immune response, enrolling other immune cells to affect neoplastic cells [61,62].

IL-22 is an IL-10 cytokine that is principally delivered by immune cells (NK cells, T-helper cells, and innate lymphocytes) [176]. Immune cells do not express IL-22 receptors [177], but IL-22 controls the tissue cells of the lungs, kidney, digestive tract, and skin [178]. The cytokine regulates growth, and the cell cycle and could be implicated in tumor genesis.

Increased concentrations of IL-22 are present in tumors such as pancreatic, colon, gastric, lung, pancreatic, and liver cancers [179]. Recent findings suggest that several cancer cell lines present IL-2-22-receptor 1 (IL-22-R1) [180].

It has also been reported that Th22 cells and IL-22 may have a role in acute lymphoblastic leukemia, acute myeloid leukemia, and myelodysplastic syndromes [181]. High IL-22 concentrations and the elevated expression of CD38 may be implicated in the synergetic stimulation of genes involved in proliferation and programmed cell death. Elevated levels are connected with poor outcome in B-CLL patients [182], and it has been reported as a prognostic biomarker in cancers [183,184].

In previous work, we examined the plasma concentrations of IL-22, IL-23, and IL-10 in patients with B-CLL. In the same patients, we also evaluated a possible correlation between IL-22 level and biological risk. There was significant variance among the concentrations of IL-22 in B-CLL patients versus healthy people. Furthermore, patients who had higher CD38 expression presented greater plasma IL-22 concentrations compared to patients with minor CD38 expression [185].

As for the mechanism by which IL-22 operates in B-CLL, it is well known that STAT3 is activated and increases the survival of B-CLL cells. IL-22R1 is linked to the principal IL-22 signaling STAT3 by the new STAT3 tyrosine-dependent recruitment and the classic STAT pathway by the STAT-binding sites. The novel mechanism works cooperatively with the C-terminal end of the interleukin 22 receptor with the coiled-coil domain. NF-kB may also act in blocking programmed cell death in B-CLL cells. Reduction of the NF-kB pathway can decrease the concentration of IL-22RAI protein expression. It was assumed that an altered expression of IL-22 may participate in the modification of several cell signaling systems, thus increasing the promotion of cell proliferation in B-CLL [63,64,65,66].

IL-22 could also act in resistance to therapy in B-CLL patients. In fact, although the exact mechanism of chemoresistance in B-CLL is not well defined, one of the principal elements could be an alteration of programmed cell death via an increase of antiapoptotic proteins, such as cytokines. B-CLL cells generally present numerous transporter proteins implicated in multidrug resistance, and an increased concentration of P-glycoprotein (P-gp) expression is a characteristic of B-CLL cells. Jamroziak et al. demonstrated that a silent polymorphism at position 3435 of the *MDR1* gene alters P-gp activity in B-CLL cells [186].

IL-23 is a cytokine of the IL-6 superfamily that is implicated in tissue remodeling and in connecting adaptive and innate immunity [187]. It is a heterodimeric cytokine constituted of a p19 subunit and a p40 subunit. IL-23 is also implicated in the immune response against tumor via the action of the IL-23 receptor (IL-23R) [188,189,190]. The receptor is made of two parts: the IL-12R1 chain (the same as IL-12R) and a particular IL-23R subunit. Only early B lymphocytes, germinal center B cells, and plasma cells have a functional IL-23R [191,192]. In tumor cells, the IL-23R molecule is present on myeloma cells, follicular lymphoma, acute lymphoblastic leukemia cells, and diffuse large B cell lymphoma cells [193,194].

The IL-23R/IL-23 axis was studied by Cutrona et al. They observed that the cells of patients affected by an early stage of B-CCL with a worse prognosis had a defective version of the IL-23R complex lacking the IL-12R1 chain. Cells with the incomplete form of the receptor could be stimulated to present the complete form if cultured with T cells or CD40L+ cells. B-CLL cells stimulated in this environment generated IL-23. This result indicates the presence of an autocrine/paracrine loop stimulating B-CLL cell growth. Interfering with the IL-23R/IL-23 pathway using an anti-IL-23p19 antibody was efficient in avoiding the start of the disease, suggesting possible therapeutic approaches [67].

IL-33 is a cytokine that regulates cytokine generation in type 2 innate lymphoid cells, Th2 lymphocytes, eosinophils, NK cells, basophils, and invariant natural killer T cells [195].

In previous work, we studied the concentrations of IL-33 in B-CLL patients. We also examined IgVH gene analysis as well as CD 38 and ZAP-70 expression. In our study, there was a relevant decrease of Il-33 in B-CLL patients compared to healthy subjects [196].

This reduction might be implicated in the T-cell alteration of B-CLL patients. IL-33, in fact, seems to control Th2 response. Podhorecka et al. [197] examined the Th1/Th2 balance in B-CLL patients and demonstrated the dominance of Th1 cells and T cell-mediated immunity that changed toward Th2 in the course of disease evolution. The decrease in plasma concentration of IL-33 might also explain the decreased Th2 response detected in these patients. Additionally, a study reported a positive link between IL-33 levels and CD3 expression and demonstrated that a minimal expression of CD3γ and ε and ζ chain genes, together with the FcεRIγ gene, exists in B-CLL patients [198]. Lastly, the study identified an inverse relationship between IL-33 concentration and CD20 expression: the concentration of IL-33 influences the expression of CD20. It could be due to a direct effect of the cytokine or to a different state. Nevertheless, the suggestion that this B-CLL therapy is capable of normalizing serum levels of the cytokine is very interesting. On this basis, we can speculate that there is a primitive effect in B-CLL on cytokine concentration [198].

TNF-α is constitutively generated by B-CLL cells, and it may operate as an autocrine element for their proliferation [73,199]. Furthermore, in B-CLL patients, TNF-α serum concentrations and soluble TNF-α receptor (sTNFR) concentrations are augmented, and correspondence with leukemia progression has been revealed. 

Data suggest that TNF-α is an essential element in the programmed cell death resistance of neoplastic lymphocytes in B-CLL. A research study offered proof of the effect of the tumor necrosis factor G/A (TNFG/A) genotype and A alleles on the propensity for leukemia, since a correlation of LT-alphaG/G genotype with CLL was described. The examined single-nucleotide polymorphism (SNP) controls the action of alkaline DNase in B-CLL patients, and the polymorphism may regulate the predisposition of B-CLL cells to programmed cell death by way of DNase activity [200].

It has been postulated that increased concentrations of TNF-α and sTNFR can be considered markers of outcome in lymphoma patients [201]. Ferraioli et al. reported a link between TNF-α plasma concentration and the severity of B-CLL [202]. High TNF-α concentrations are suggestive of aggressive leukemia, thus suggesting an action in B-CLL evolution. TNF-α was reported to have autocrine action in B-CLL [203,204,205,206,207]. Blocking TNFR signaling by interference with etanercept, a recombinant TNFR-2 product, combined with the anti-CD20 antibody rituximab, caused effective remission in refractory patients without 17p deletion [208].

Other studies demonstrated that TNF stimulates the growth of leukemic B cells and has action in the progression of B-CLL [68,69].

IFN-γ is the main macrophage-activating cytokine and has significant roles in innate immunity and adaptive cell-mediated immunity. The principal cellular origin of IFN-γ is Th1 cells [73]. B-CLL cells may also deliver IFN-γ and express IFN-γ receptors; thus, it has been postulated that an autocrine pathway participates to block programmed cell death [209]. In vivo, interferon γ is the most conspicuous plasma cytokine in low-stage B-CLL [210]. However, the in vitro lymphocyte delivery of IFN-γ is significantly decreased in B-CLL patients. B-CLL lymphocytes present augmented rates of spontaneous programmed cell death after incubation for 24 h. Remarkably, the augmented spontaneous programmed cell death of B-CLL lymphocytes is followed by reduced IFN-γ production. The stage of leukemia did not change the spontaneous programmed cell death in vitro [211].

## 2. Future Perspectives

In the future, new and more reliable investigations will allow us to better define the cytokine patterns in B-CLL patients.

For instance, gene expression profiling (GEP) of stage 0/I B-CLL patients demonstrated increased Frizzled 3/ROR-1 receptor tyrosine kinase (FLT-3 RTK) and chemokine receptor CXCR3 G-protein coupled receptor, fibromodulin, transforming growth factor (TGF) β, TGF βRIII, IFN-γ, CSF3, insulin-like growth factor binding protein 4, and SMAD2. Moreover, in order to establish why B-CLL cells proliferate inadequately in culture, B-CLL patient cells were cultured, and GEP displayed increased chemokine (C-C motif) ligand 2 (CCL2), CXCL3, and CXCL5, which may enroll immune cells [212].

Furthermore, the cytokine investigation of B-CLL patients could open novel therapeutic possibilities. B-CLL is incurable with standard treatment, so creating new approaches to increase immune activity deserves supplementary research. Emergent treatments such as chimeric antigen receptor-modified T cells (CART) and B-CLL vaccines are under clinical examination [213,214].

A different therapeutic approach has been attempted by other researchers. Folate conjugated to immunoglobulin (F-IgG) attracts innate immune system cells with Fc receptors to folate receptor-presenting cancer cells. The identification of F-IgG by NK cell Fc receptors caused phosphorylation of the extracellular signal-regulated kinase (ERK) transcription factor. The destruction of KB tumor cells by NK cells increased up to five-fold after treatment with F-IgG, which is a strategy that is synergistically amplified by the administration of other interleukins such as IL-2, IL-12, IL-15, or IL-21. F-IgG also augmented the lysis of B-CLL cells by autologous NKs. NK cells significantly increased the production of IFN-γ and Macrophage Inflammatory Protein (MIP)-1α once stimulated by F-IgG-coated KB target cells while exposed to the NK cell-activating cytokine IL12. F-IgG-coated targets similarly activated Fc receptor (FcR)-mediated T cell chemotaxis and monocyte activity. Research performed on the animal leukemia model proved the intratumoral localization and antitumor action of F-IgG, as well as improvement of its actions by IL12. Thus, F-IgG can stimulate an immune response against FR-positive tumor cells that is controlled by NK cells and can be increased by cytokine administration [215].

Deng et al. produced a fusion cytokine called GIFT4 as a result of the combination of human GM-CSF and IL-4. B-CLL cells were exposed to GIFT4. The cytokine amplified the expression of stimulatory molecules such as CD40, CD80, CD86, and adhesion molecule CD54. The exposed B-CLL cells produced IL-1β and IL-6. Moreover, GIFT4 therapy provoked a JAK1-, JAK2-, and JAK3-mediated hyperphosphorylation of STAT5 in B-CLL cells and increased the growth of IFN-γ-generating CD314+ cytotoxic T cells, which were able to lyse B-CLL cells. Furthermore, the use of GIFT4 cytokine amplified the recruitment of T cells in Nonobese diabetic/severe combined immunodeficiency (NOD-scid) IL2Rγ-null immune-deficient mice adoptively pre-transferred with mononuclear cells from B-CLL patients [216].

A different therapeutic strategy is the capacity of altering cytokine-caused signaling.

For instance, TNF-α-provoked NF-κB stimulation was demonstrated to be reduced by wogonin, a natural flavonoid, causing a modification of TNFR-1 signaling toward programmed cell death induction [217]. Several in vitro and in vivo studies demonstrated that wogonin has antioxidant and anticancer actions [218]. In B-CLL patients, increased concentrations of TNF-R1 were correlated with poor clinical outcomes. The activation of TNF R-1 with TNF-augmented NF-κB behavior and the survival of B-CLL cells was diminished by wogonin. The surprising effect of wogonin was deepened in an animal model after the adoptive transfer of E/T cell leukemia 1 leukemic cells. Wogonin administration stopped leukemia onset when imparted soon after transplantation. The treatment of leukemia caused a reduced expression of TNF R-1 on B-CLL cells and their passage to blood. Affecting TNF R-1 signaling is an interesting option for B-CLL therapy [219].

Similarly, the use of immunomodulatory drugs could normalize the immunological status of B-CLL patients. After 15 cycles of therapy, responder patients presented decreased lymphocytes, activated CD4+ T cells generating IFN-γ, IL-2, or TNF-α, and Treg cells [220].

Finally, a particular field of interest is the relationships between oxidative stress, cytokines, and B-CLL. In fact, patients with B-CLL have profound oxidative alteration [221,222].

Oxidative stress increases the spontaneous programmed cell death of B-CLL lymphocytes, and this could be exploited for therapeutic purposes [223]. An inflammatory environment is produced in survival-sustaining B-CLL cultures, and this might maintain the persistence and proliferation of leukemic cells. Moreover, in the study, the authors recognized new genes, particularly chemokine (C-C motif) ligand 2, and pathways, particularly nuclear respiratory factor-2 (NRF2)-mediated oxidative stress response signaling. Their findings suggest that CCL2 and possibly other inflammatory cytokines are implicated in B-CLL cell survival in vitro, and they might be relevant in vivo as well. Intervention with these mechanisms could interfere with the survival of neoplastic cells [224].

## 3. Conclusions

B-CLL is characterized by increased monoclonal B lymphocytes, whose proliferation and persistence necessitate endogenous and exogenous stimulation elements [225,226]. Significant progress has been made in comprehending this cross-talk [227,228], with clinical and translational research confirming the actions of several cytokines and chemokines in the complicated activation of B-CLL cells within the microenvironment [229,230]. Nevertheless, as several cytokines augmented in diverse B-CLL microenvironments have multiple actions, with coinciding as well as antagonistic activities, establishing a unified outline of coordinately produced cytokines that may contribute to B-CLL is indispensable [231]. In B-CLL patients, an unsupervised hierarchical bicluster evaluation recognized three diverse clusters (CLs) of extremely interrelated but differentially produced cytokines: CL1 (IL-15, IL-12, IFN, CXCL9, CXCL10, CXCL11, CCL3, CCL4, and CCL19), CL2 (IL-6, IL-8, GM-CSF, and TNF), and CL3 (IFN, IL-1, IL-4, IL-15, and IL-17). Permutation scores mixing the expression of CL1/CL2 or CL1/CL3 showed a strong relationship with time to first therapy and overall survival. Patients with the poorest outcome had elevated CL1 and decreased CL2 or CL3 concentrations. Multivariate analysis demonstrated that CL1/CL2 score and IgVH mutation status were unconnected prognostic markers for time to first treatment, while CL1/CL3 score and IgVH were separate indicators for overall survival. These results suggest the importance of multi-cytokine evaluation and novel therapeutic approaches for B-CLL patients that is planned to decrease CL1 and augment CL2/CL3 cytokines [95].

Moreover, cytokines should be studied in both the bone marrow and lymphoid tissues, because concentrations in serum may be different from those at such sites.

**Figure 1:**
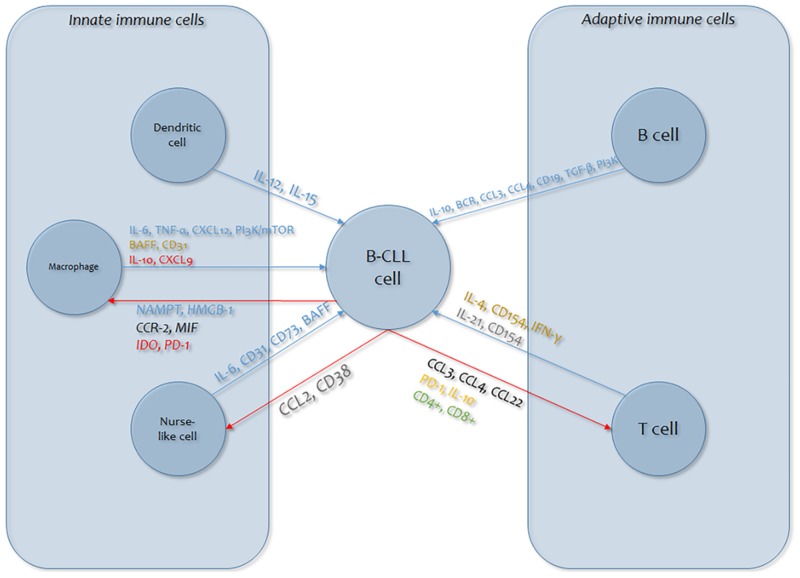
Graphic overview of the mechanisms of cytokine involvement in B-cell lymphocytic leukemia (B-CLL) cellular dynamics (blue arrows: effects of immune cells on B-CLL cells; red arrows: B-CLL cell responses). Cytokines in plain text are those involved in the effects on B-CLL cells (blue: signaling; red: immunosuppression; gold: survival; gray: proliferation); cytokines in italics are those involved in the B-CLL cell response (green: expansion; orange: exhaustion; black: chemoattraction; red: immunosuppression; blue: pro-tumor skewing; gray: immune evasion). CCL: chemokine (C-C motif) ligand; CD38: Custer of Differentiation; IL: Interleukin PD-1: Programmed cell death protein 1; MIF: Macrophage migration inhibitory factor; CCR-2: C-C chemokine receptor type 2; NAMPT: Nicotinamide phosphoribosyltransferase; HMGB: High Mobility Group Box; CXCL: chemokine (C-X-C motif) ligand; BAFF: B-Cell Activating Factor; TNF: Tumour Necrosis Factor.

**Figure 2:**
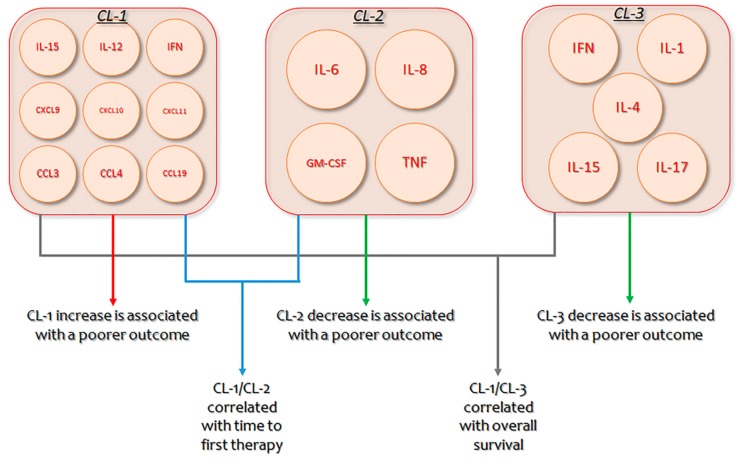
Clusters of identified cytokines, their respective interplay, and their impact on Chronic lymphocytic leukemia (CLL) prognosis. CL: Cluster; IFN: interferon; GM-CSF: Granulocyte-Macrophage Colony-Stimulating Factor.

Finally, our understanding of the intricate cross-talk between the several sorts of bystander cells (such as myeloid suppressive cells, macrophages, dendritic cells) and B-CLL cells is inadequate (Figure 1). Bystander cells give B-CLL cells helpful functions, but it has also become evident that B-CLL cells dynamically participate in the formation of a favorable tumor microenvironment. Several regulators are implicated in these communication dynamics, primarily cytokines, which are implicated in cell persistence and growth and in immunoregulation (Figure 2) [232,233].

The capability to block such mediator connections in vivo may be manipulated to improve already known therapies for B-CLL.

## Figures and Tables

**Table 1:** Effects of cytokines on the cellular dynamics of B-cell lymphocytic leukemia (B-CLL).

Cytokine	Action	Mechanism	Reference
IL-2	Protumoral	Reduced apoptosis	[23]
		Increased growth	[24]
IL-4	Protumoral	Reduced apoptosis	[25,26,27,28,29,30,31,32]
		Stimulation of STAT	[33]
		Increase miR-21-5p	[34]
		iNKT alteration	[35]
IL-6	Protumoral	Stimulation of STAT3	[36]
		Stimulation of NF-kB	[36]
		Modulation of VEGF	[37,38]
	Antitumoral	Action on Toll-like receptor	[39,40]
IL-8	Protumoral	Increase of BCL-2	[41,42]
		Angiogenesis	[43,44]
IL-9	Protumoral	Increased proliferation	[45]
		Decreased apoptosis	[46]
		Increased JAK/STAT	[47,48]
IL-10	Protumoral	Increased proliferation	[49,50]
	Antitumoral	Action on NK cells	[51,52]
IL-17	Protumoral	Increased IL-6	[53]
	Antitumoral	Action on immunity	[54]
IL-21	Antitumoral	Increased apoptosis	[55,56,57,58,59,60]
		Increased proliferation of cytotoxic T cells	[61,62]
IL-22	Protumoral	Stimulation of STAT3	[63,64,65,66]
		Reduced apoptosis	[63,64,65,66]
IL-23	Protumoral	Unclear	[67]
TNF-alpha	Protumoral	Increased proliferation	[68,69]

STAT: Signal Transducer and Activator of Transcription; INKT: invariant Natural Killer; VEGF: Vascular Endothelial Cell Growth Factor; BCL-2: B-cell lymphoma 2; NK: Natura Killer; IL: Interleukin; TNF: Tumour Necrosis Factor.
